# Severe Type of Minocycline-Induced Hyperpigmentation Mimicking Peripheral Arterial Occlusive Disease in a Bullous Pemphigoid Patient

**DOI:** 10.3390/antibiotics8030093

**Published:** 2019-07-16

**Authors:** Meng-Yu Wu, Yueh-Tseng Hou, Giou-Teng Yiang, Andy Po-Yi Tsai, Ching-Hsiang Lin

**Affiliations:** 1 Department of Emergency Medicine, Taipei Tzu Chi Hospital, Buddhist Tzu Chi Medical Foundation, New Taipei 231, Taiwan; 2 Department of Emergency Medicine, School of Medicine, Tzu Chi University, Hualien 970, Taiwan; 3 Department of Medical Research, Buddhist Tzu Chi General Hospital, Hualien 970, Taiwan

**Keywords:** hyperpigmentation, minocycline, peripheral arterial occlusive disease, bullous pemphigoid

## Abstract

Minocycline is a tetracycline group antibiotic that is known to cause significant antibacterial and anti-inflammatory effects. Minocycline has been widely used to treat systemic infection, acne, dermatitis, and rosacea. However, various dose-related side effects of hyperpigmentation in whole body tissues have been reported. Three main types of minocycline-induced hyperpigmentation have been identified. In rare severe hyperpigmentation cases, drug-induced hyperpigmentation can mimic local cellulitis or peripheral arterial occlusive disease (PAOD). These processes require different therapeutic strategies. Therefore, early diagnosis is extremely important for physicians to determine the etiology of the hyperpigmentation, and subsequently discontinue the minocycline if indicated. We describe a rare case presenting a severe form of type III minocycline-induced hyperpigmentation mimicking peripheral arterial occlusive disease in a bullous pemphigoid patient.

## 1. Introduction

Minocycline is a highly lipophilic antibiotic in the tetracycline group of antibiotics that exhibits both antibacterial and anti-inflammatory effects [1]. The high penetration capability of minocycline in all body tissues contributes to its high efficiency in controlling infections [2,3]. Minocycline is also used to treat acne, rosacea, and dermatitis, owing to its anti-inflammatory effects, and has been widely used in dermatologic practice. Minocycline use can be complicated by dose-related gastrointestinal side effects, such as nausea and vomiting. In addition, minocycline has been reported to induce hyperpigmentation of whole body tissues, including the skin, mucosa, teeth, conjunctiva, nail beds, and bones.

Drug-induced hyperpigmentation occurs with minocycline, chloroquine, amiodarone, and cyclophosphamide via an increased deposition of melanin in the epidermis and dermis, especially in photo-exposed skin. Certain symptoms, including local edema, heat, skin wound, or formation of wound exudate, are similar to those of cellulitis and peripheral arterial occlusive disease (PAOD). In severe hyperpigmentation cases, the clinical picture may mimic that of cellulitis or PAOD, which warrant different therapeutic strategies. Therefore, early diagnosis is imperative for physicians to recognize the etiology and discontinue minocycline, if necessary.

In this article, we present a rare case of a patient with severe type minocycline-induced hyperpigmentation, combined with atypical PAOD symptoms. Minocycline-induced hyperpigmentation rarely mimics PAOD or cellulitis. In the bullous pemphigoid population, bullous rupture may induce cellulitis or present as a chronic skin ulcer, delaying timely diagnosis. In this report, we discuss the clinical features and skin pattern of minocycline-induced hyperpigmentation. We also highlight that minocycline-induced hyperpigmentation can mimic other acute processes, confounding the diagnosis. This can lead to the persistence of the side effects if minocycline is not discontinued.

## 2. Case Presentation

This case report was approved by the Institutional Review Board of Taipei Tzu Chi Hospital, Buddhist Tzu Chi Medical Foundation.

**Figure 1:**
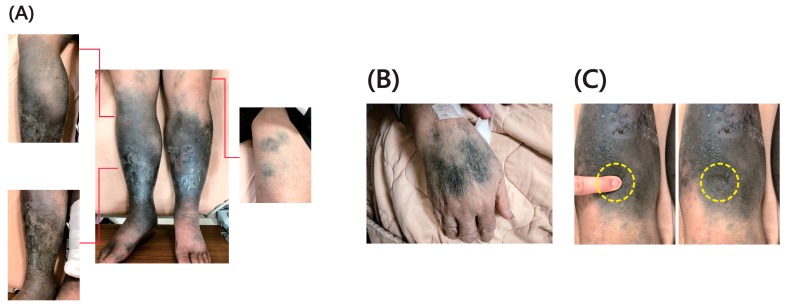
(**A**) Severe, dark black discoloration patches with local scarring of hyperpigmentation noted on bilateral lower legs. (**B**) The non-palpable and non-pruritic bluish discoloration patches on arms. (**C**) There was no local swelling, heat, tenderness, or erythematous changed at bilateral lower legs. The compression test on bilateral lower legs showed no blanching with pressure.

A 68-year-old male presented with progressive bluish discoloration of his bilateral lower legs for several months. His past medical history included bullous pemphigoid and chronic obstructive pulmonary disease with recurrent pneumonia. Two years prior, he was diagnosed with bullous pemphigoid complicated by bilateral lower leg cellulitis. He had been taking prednisolone 5 mg orally three times a day and minocycline 100 mg orally twice a day for the previous two years. This was to control the pemphigoid as a chronic suppressive treatment. The patient was noncompliant with medication and treatment, resulting in lower leg erythematous changes with local swelling. The patient then self-treated with antibiotics. Initially, the bluish discoloration was present only on one leg but eventually progressed to both legs. The patient visited other hospitals, and a diagnosis of PAOD and deep vein thrombosis was considered. This was due to the presence of symptoms including local skin ulcers, bluish skin, symmetric involvement, and tissue infection. Apixaban was administered, but the bluish discoloration persisted. The patient developed bluish-grey macules and patches that progressed on bilateral forearms and hands. When he visited our emergency department, a review of medication history revealed only minocycline administration associated with pigmentation. There was no other known cause in the patient’s history to explain the symptoms; the patient had no contact history of heavy metals, such as lead, mercury, silver, bismuth, and arsenic. There was no medicine record of administration of chloroquine, amiodarone, or cyclophosphamide. On admission, his temperature was 37.1 °C, his blood pressure was 126/71 mm Hg, and his heart rate was 96 beats/min. On physical examination, severe, black discoloration patches with local scarring were noted on bilateral lower legs (Figure 1A). There was no local swelling, heat, tenderness, or erythematous changes on bilateral lower legs. There were non-palpable and non-pruritic bluish discoloration patches on his arms (Figure 1B). A compression test performed on bilateral lower legs was negative, showing no blanching with pressure (Figure 1C).

**Figure 2:**
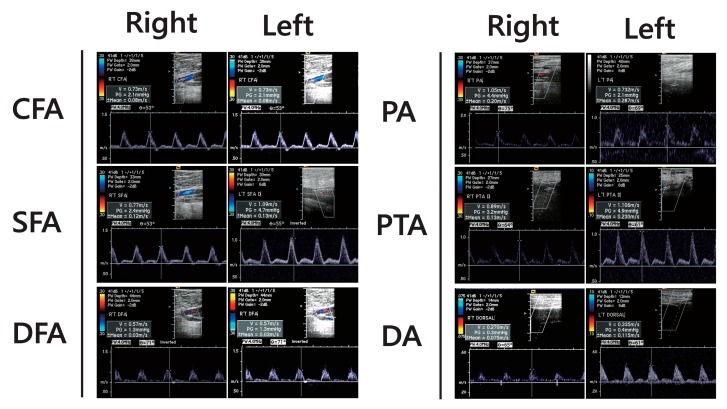
The doppler ultrasound of extremities revealed no significant decrease of blood flow or abdominal atherosclerosis in vessels. Right: right leg; Left: left leg; CFA: common femoral artery; SFA: superficial femoral artery; DFA: deep femoral artery; PA: popliteal artery; PTA: posterior tibial artery; DA: dorsalis pedis artery.

A doppler ultrasound of bilateral extremities and pressure recording was done to rule out PAOD and revealed no significant atherosclerosis in bilateral lower extremities (Figure 2).

The clinical symptoms and laboratory workup were not compatible with a diagnosis of deep vein thrombosis. We diagnosed the patient with a rare severe form of type III minocycline-induced hyperpigmentation. The antimicrobial medication was stopped immediately.

## 3. Discussion

Minocycline is a tetracycline antibiotic used to treat a number of conditions. It exhibits high tissue penetration capability, and treats infection against both gram-positive and gram-negative bacteria. Minocycline is also an alternative treatment for actinomycosis, amebiasis, anthrax, *Clostridium* spp., *Listeria monocytogenes, Neisseria meningitides,* and sexually transmitted infections, when penicillin is contraindicated [4,5,6,7]. Minocycline has also long been used to treat acne vulgaris and rosacea via anti-apoptotic, anti-inflammatory, and immune-modulatory effects [8]. Cutaneous hyperpigmentation is a reported adverse effect of minocycline administration. In a study reported by Dwyer et al. [9], eight (14.8%) of fifty-four patients taking minocycline for acne or rosacea developed hyperpigmentation, with a mean duration of 17 months. The average cumulative dose was 47 g. In a study by Hanada et al. [10], 156 (54%) of 291 patients receiving long-term minocycline therapy for orthopedic infections developed hyperpigmentation, with a mean duration of 1.5 years and mean cumulative dosage of 107.3 g. Most minocycline-induced hyperpigmentation was type II (87.8%). The severe type of hyperpigmentation was rare, accounting for 1.9% of such cases. In a placebo-controlled study of 44 rheumatoid arthritis patients, three patients developed minocycline-induced hyperpigmentation in the control group compared to zero patients in the placebo group [11]. No accurate incidence of minocycline-induced hyperpigmentation from a large cohort study has been reported in the current literature. Gordon and colleagues [12] stated that only 77 minocycline-induced hyperpigmentation events were reported to the manufacturer since 1985, while 55 million doses of minocycline have been prescribed. Although minocycline-induced hyperpigmentation is uncommon, compared to other types of drug-induced hyperpigmentation it still has a higher incidence. These data were confirmed in a systematic review by Krause et al. [13], which revealed a causal relationship between five classes of drugs and hyperpigmentation. These include prostaglandins, minocycline, phenothiazine, nicotine, and antimalarial drugs.

Although several studies have focused on detailing the mechanism of minocycline-induced pigmentation, it remains unclear. Several theories have been proposed for hyperpigmentation. In one in vitro study by Sato E. et al. [14] using B16 melanoma cells as model, it was revealed that the expression of mRNA of tyrosinase and tyrosinase-related protein (TRP)-1 and 2 were increased after treatment with minocycline. In addition, the p38 inhibitor inhibited the mRNA expression of tyrosinase, suggesting that minocycline-induced melanogenesis occurs via a p38 signaling pathway. In one study, Sato S. et al., through ultrastructural and X-ray microanalytical observations, identified an important role of siderosis in the pathogenesis of minocycline-induced hyperpigmentation [15]. Siderosis may be minocycline-induced from local microhemorrhage due to trauma [16]. In a study by Okada et al. [17], a series of biopsies were performed in a patient with minocycline-induced hyperpigmentation. Their findings supported that minocycline chelated with iron-forming insoluble complexes in pigment granules.

Minocycline-induced hyperpigmentation may involve several tissues, such as the skin, thyroid sclera, nail, oral mucosa, teeth, bone, trachea, and tympanic membrane [1,18,19,20,21]. In clinical classification, the minocycline-induced hyperpigmentation was divided into three main types (Table 1). In type I, the pigmentation color is blue-gray to black, and is located mainly on the previous inflammation area or scarring of skin on the face [22]. Type I pigmentation was not associated with either doses or duration of minocycline intake [9]. There may be a delayed presentation of up to one week after administration of minocycline. Type II pigmentation is blue-gray in color and is located on forearms, hands, or legs. Type III pigmentation presents with diffuse muddy-brown discoloration on photo-exposed skin [23]. In types II and III pigmentation, there is an association between the duration of minocycline intake and the duration of pigmentation reported, with the median duration lasting more than 9 months with cumulative doses [10,24,25,26].

In severe cases of both type II and III hyperpigmentation, the clinical presentation may mimic PAOD of atherosclerosis. Atherosclerosis leads to a decrease in arterial blood flow, causing distal limb ischemia. This hypoxic state promotes tissue damage and chronic ulcer formation [27]. Venous stasis and recurrent tissue necrosis may lead to pigmentation on bilateral legs. Likewise, recurrent cellulitis is also known to promote local hyperpigmentation. These similar symptoms and clinical presentations can be difficult for a physician to diagnose and initiate timely intervention. In our case, the atypical symptoms, including dark hyperpigmentation and recurrent local wounds, were mimicking those of PAOD, especially in a bullous pemphigoid patient. Atypical PAOD was easy to exclude with doppler ultrasound. The doppler ultrasound of an extremity is an effective tool to rule out PAOD with sensitivity of 79.7% and specificity of 79.2% for ≥70% lesions [28]. The patient’s medical history is very useful when considering a diagnosis of drug-induced hyperpigmentation. The delayed diagnosis of minocycline-induced hyperpigmentation may lead to the patient receiving unnecessary antibiotic treatment for cellulitis, or anticoagulation treatment for PAOD. Additionally, the minocycline-induced hyperpigmentation is cosmetically disfiguring and may cause significant effects on self-confidence and self-esteem [29]. Without timely intervention, hyperpigmentation may persist for several months or even years after discontinuing minocycline therapy [1,30]. Laser treatments may provide effective clearing of the minocycline-induced hyperpigmentation in severe cases [29,31].

## 4. Conclusions

In conclusion, this article presented a rare case of a patient with severe type III minocycline-induced hyperpigmentation combined with atypical PAOD symptoms. We also highlighted that minocycline-induced hyperpigmentation may mimic PAOD or cellulitis, leading to the persistence of the minocycline side effect. It is important for physicians to discontinue minocycline in a timely fashion if appropriate and switch to an alternative treatment.

## Figures and Tables

**Table 1:** The characteristics of three main types of minocycline-induced hyperpigmentation.

Type	Skin Color	Pigmentation Pattern	Localization	Histological Finding
Type I	Blue-gray to black	Circumscribed	Face, inflammation area	Pigment location in dermis
Type II	Blue-gray	Circumscribed	Legs, forearms, hands	Pigment location in dermis/hypodermis
Type III	Muddy brown	Diffuse	Sun-exposed skin	Pigment location in dermis/epidermis
